# Patient Preferences and Satisfaction With Refractive Error Correction Methods: A Cross-Sectional Study

**DOI:** 10.7759/cureus.90220

**Published:** 2025-08-16

**Authors:** Ithar M Beshtawi, Shorooq Faqarah, Heba Abdul-Fatah

**Affiliations:** 1 Optometry Department, Faculty of Medicine and Allied Medical Sciences, An-Najah National University, Nablus, PSE

**Keywords:** contact lenses, preference, refractive correction, refractive surgery, satisfaction, spectacles

## Abstract

Purpose: Uncorrected refractive error (RE) is a leading cause of visual impairment. Correction through glasses, contact lenses (CL), or refractive surgery (RS) is crucial to prevent functional and quality-of-life limitations. This study aimed to evaluate the factors affecting the patient preferences regarding the correction methods.

Materials and methods: A cross-sectional study was employed, including 451 patients (346 females; mean age: 24.30 ± 6.2 years) with RE corrected by either glasses, CL, or RS. A structured questionnaire was used that included demographics, clinical characteristics, preferences, and satisfaction.

Results: The demographics and the extent of refractive error significantly influenced the selection of correction methods (p = 0.00). Professional recommendations (66.0%) and cost considerations (47.1%) are the main motivations for acquiring spectacles. They avoided CL due to side effects (69.9%) and maintenance difficulties (54.9%). In the CL groups, the primary motivation was cosmesis (82.2%, p > 0.05), while they avoided glasses due to inconvenience (59.6%). Neither group chose RS due to associated side effects (45.8% and 55.5%, respectively) and insufficient information regarding the procedure (39.2% and 47.9%, respectively). The primary motivations for RS are the inconvenience associated with glasses and CLs (73.3%) and aesthetic concerns (67.1%). They avoided spectacles due to inconvenience (70.4%) and unsatisfactory cosmetics (67.8%), whereas CLs were avoided due to concerns about potential side effects (67.1%) and maintenance challenges (68.4%). The RS group exhibited a significantly (P<0.001) higher satisfaction rate at 88.8%, compared to the glasses group at 71.9% and the CL group at 63.0%. Discomfort (30.1%) and cosmesis (29.4%) significantly influenced dissatisfaction with glasses (p = 0.00). Related side effects (60.3%), limitations during swimming (56.2%), maintenance difficulties (43.2%), and cost (15.1%) significantly influenced dissatisfaction with CL (p = 0.00).

Conclusion: Based on self-reported satisfaction scores, RS users expressed the highest levels of satisfaction, followed by CL and spectacle users. Patient preferences appeared to be influenced by cost, comfort, and cosmetic concerns.

## Introduction

Global research over the past two decades has documented a significant rise in the prevalence of refractive error (RE), especially myopia [1]. This increase may be associated with excessive near work due to continuous usage of digital devices at an early age [2]. Additionally, almost all individuals will have presbyopia, resulting in diminished near vision [1]. Uncorrected refractive error (URE) is regarded as the leading cause of visual impairment in both children and adults, representing approximately 50% of instances of moderate to severe visual impairment worldwide; this percentage may increase in the future [1]. REs can lead to restricted educational [3] and employment opportunities [4], in addition to a general decline in quality of life [5]. These factors emphasize the necessity of addressing these concerns through various corrective methods, such as eyeglasses, contact lenses (CL), and refractive surgeries (RS) [6].

Individuals may choose a corrective method while avoiding others based on personal preferences [7-9]. Eyeglasses are widely acknowledged as the most cost-effective option [10]. CLs enhance cosmetic appearance [7], and RS has gained popularity due to factors such as improved visual outcomes, better aesthetics, and an overall enhanced quality of life [11]. On the other hand, each correction method has some drawbacks that might deter patients from using it. For instance, some people perceive glasses as an uncomfortable option [8], while others have concerns about the associated side effects and expenses of CL [12,13] or RS [14,15]. Additionally, variations in the socioeconomic status and cultural prospects among different countries also affect the patients' preferences for correction methods. Other factors that may impact an individual's selection among available corrective methods include advancing technology, changes in fashion, cosmetic trends, lifestyle trends, environmental demands, media representation, peer influence, and professional recommendations, all of which fluctuate over time.

There have been no prior studies examining the factors influencing the preferences of ametropic patients regarding correction methods in Palestine. This study aims to evaluate and compare patients' preferences, satisfaction levels, and the factors influencing their choice of refractive correction methods, including spectacles, CL, and RS, among a diverse patient population. The findings of this research will help to ensure that eye care practitioners meet the needs of their patients to provide them with the most professional personalized care.

## Materials and methods

Participants of both genders, aged 18 years and older, with a RE corrected by eyeglasses, CL, or RS were eligible to participate. Patients with identified ocular disease (e.g., keratoconus, glaucoma, or retinal disorders) or a prior history of ocular surgery other than refractive procedures were excluded from this study. Participants were recruited using a convenience sampling approach. Invitations were distributed through announcements on social media platforms and flyers posted at optometry practices and ophthalmic hospitals. This study employed a questionnaire that was developed by the research team based on findings and constructs from a previous study [7] aimed at identifying the factors that influence patients' decisions regarding RE correction methods. To assess the content validity, the questionnaire was initially pilot-tested with 15 patients who corrected their RE using glasses, CLs, or RS. Based on their feedback and following a review by two experts in the field, the questionnaire was revised to enhance clarity and comprehensibility. The final version of the questionnaire was then used for data collection, and responses from the initial 15 participants were excluded from the analysis.

Participants were interviewed by a trained researcher to complete the questionnaire. Initially, participants were requested to provide demographic information, such as their age, gender, and educational level. Participants were then asked about their eye condition and the type of correction they use, which could be eyeglasses, contact lenses (either soft or hard), or laser surgery options like laser-assisted in situ keratomileusis (LASIK), photorefractive keratectomy (PRK), or Phemto-small incision lenticule extraction (SMILE). Participants were also asked about the medical center they visit (whether it is an ophthalmology or optometry practice) and the factors that influenced their choice. These factors included the center's reputation, professional advice, availability of diverse treatment options, prior experiences, negative experiences at other facilities, advertising, cost, and location. Satisfaction with the corrective method was evaluated through a five-point Likert scale, with responses ranging from 0 (completely dissatisfied) to 4 (completely satisfied).

Then the participants were inquired about the factors influencing their choice. These factors include peer recommendations, professional advice, work-related requirements, advertising influence, concerns regarding CL or RS, cosmetics, and cost. They were also requested to identify factors that contributed to dissatisfaction with their choice. Factors considered included cosmetic considerations, potential side effects, discomfort, frequent loss or maintenance challenges, high cost, and limitations on sports activities. Then, the rationale for excluding the other two corrective methods was explored. The variables under consideration included concerns about potential side effects, discomfort, cosmetic considerations, work-related requirements, unfavorable professional recommendations or advice from friends or relatives, negative publicity or reputation, social perceptions, insufficient information, cost considerations, frequent loss or handling difficulties, and limitations in sports activities.

Ethical consideration

This research was conducted in adherence to the principles outlined in the Declaration of Helsinki. Ethical approval was obtained from the Institutional Review Board (IRB) at An-Najah National University (Ref: Med. Jan 2023/11). All participants provided informed consent before the start of the study.

Data entry and statistical analysis were performed using the Statistical Package for the Social Sciences (SPSS), version 20.0 (SPSS Inc., Chicago, IL, USA). Descriptive statistics were reported as means and standard deviations (SDs) for continuous variables, and frequencies and percentages for categorical variables. As the data were not normally distributed, non-parametric statistical tests were applied. The chi-squared (χ²) test was used to examine associations between demographic and clinical variables, patient preferences, and satisfaction levels. To evaluate the strength of these associations, Cramér's V values were calculated and interpreted as follows: values ≤ 0.1 indicate a weak association, 0.1-0.3 a moderate association, and ≥ 0.3 a strong association. A p-value of ≤0.05 was considered statistically significant.

## Results

A total of 451 participants were interviewed, comprising 105 males (23.3%) and 346 females (76.7%). The age range was 20 to 40 years, with a mean of 24.3 years (SD = 6.2). A majority of 345 participants (76.5%) were aged between 20 and 25 years, and 390 participants (86.5%) held a bachelor's degree, as shown in Table 1. A total of 278 participants (61.6%) exhibited a spherical equivalent ranging from -0.25 to -3.00. Of these, 153 participants (33.9%) corrected their vision with eyeglasses, 146 participants (32.4%) used CL, and 152 participants (33.7%) underwent RS. A Pearson chi-square test showed a significant association between mode of correction and age groups, χ²(6, N = 451) = 142.55, p = 0.00, Cramér's V = 0.398; gender, χ²(2, N = 451) = 61.96, p = 0.00, Cramér's V = 0.371; educational level, χ²(8, N = 451) = 53.11, p = 0.00, Cramér's V = 0.343; size of RE, χ²(8, N = 451) = 30.55, p = 0.00, Cramér's V = 0.184; and the attended ocular medical center χ²(2, N = 451) = 28.52, p = 0.00, Cramér's V = 0.251.

A total of 316 participants (70.1%) attend ophthalmic centers for vision correction, while 135 individuals (29.9%) declared attending optometry practices. The primary factors influencing their selection include a good reputation (N = 369, 81.8%), preferred location (N = 341, 75.6%), good prior experience (N = 309, 68.5%), and the availability of diverse treatment options (N = 240, 53.2%). The participant's choice was only significantly influenced by the availability of diverse treatment options, χ²(1, N = 451) = 6.36, p = 0.01, Cramér's V = 0.119; and prior positive experiences χ²(1, N = 451) = 5.39, p = 0.02, Cramér's V = 0.109.

**Table 1:** Participants demographics, clinical characteristics, and ocular medical center preferences, (N = 451). Data are presented as frequency (N) and percentage (%). Significant at p < 0.05. SE: spherical equivalent, PRK: photorefractive keratectomy, LASIK: laser-assisted in situ keratomileusis, SMILE: small incision lenticule extraction.

Characteristics	Frequency (N)	Percentage (%)	Mode of correction	
			χ²(df)	p-value
Gender				
Male	105	23.30%	χ²(2) = 61.96	0.00*
Female	346	76.70%		
Age				
20-25	345	76.50%	χ²(6) = 142.55	0.00*
26-30	59	13.10%		
31-35	21	4.70%		
36-40	26	5.80%		
Education level				
Diploma	52	11.50%		
Bachelor’s degree	390	86.50%	χ²(8) = 53.11	0.00*
Higher studies degree	9	2.00%		
Refractive error (SE)				
+3.25 to +6.00	17	3.80%	χ²(8) = 30.55	0.00*
+0.25 to +3.00	33	7.30%		
-3.00 to -0.25	278	61.60%		
-6.00 to -3.25	115	25.50%		
-9.00 to -6.25	8	1.80%		
Refractive mode of correction				
Glasses	153	33.90%		
Contact lenses	146	32.40%		
Soft contact lenses	98	67.10%		
Rigid gas permeable lenses	48	32.90%		
Refractive surgery	152	33.70%		
LASIK	85	55.90%		
PRK	65	42.80%		
Phemto-SMILE	2	1.30%		
Ocular medical center				
Optical practice	135	29.90%	χ²(2) = 28.52	0.00*
Ophthalmology center	316	70.10%		
Factors influencing the choice of medical center				
Good reputation	369	81.80%	χ²(31) = 76.86	0.61
A professional advice	185	41.00%		
Variety of treatment options	240	53.20%		
Good previous experience	309	68.50%		
Bad experience elsewhere	87	19.30%		
Advertisement	76	16.90%		
Cost	148	32.80%		
Location	341	75.60%		

A total of 153 participants utilized eyeglasses for RE correction, comprising 35.3% males (N = 54) and 64.7% females (N = 99). The predominant age group was 20-25 years, accounting for 88.2% (N = 135) of the sample. Table 2 presents the reasons for this group's participants' preference for glasses and their avoidance of alternative options. Participants primarily preferred glasses over alternative corrective options for various reasons: professional recommendations (N = 101, 66.0%), cost considerations (N = 72, 47.1%), and work-related requirements (N = 74, 48.4%), in addition to concerns about using CL (N = 74, 48.4%) or undergoing RS (N = 58, 37.9%). In this group, 71.9% (N = 110) exhibited satisfaction with their glasses, while only 8.4% (N = 11) reported dissatisfaction. Several factors contributed to unhappy experiences with glasses, including discomfort during wear (N = 46, 30.1%), limitations in sports activities (N = 46, 30.1%), and cosmetic appearance (N = 45, 29.4%). A Pearson chi-square test showed a significant association between satisfaction and cosmetic appearance, χ²(2, N = 153) = 36.37, p = 0.00, Cramér's V = 0.49, and discomfort, χ²(2, N = 153) = 15.61, p = 0.00, Cramér's V = 0.32. No significant association was found with the other factors (p > 0.05). Participants were asked to provide reasons for avoiding CL: side effects (N = 107, 69.9%), challenges related to lens hygiene and care (N = 84, 54.9%), and discomfort associated with touching the eye (N = 78, 51.0%). Additionally, participants avoided opting for RS due to its potential side effects (N = 70, 45.8%), a lack of sufficient information regarding the procedure (N = 61, 39.9%), and general worries about the surgery itself (N = 60, 39.2%). In this group, cost considerations significantly influenced the selection of eyeglasses χ²(3, N = 153) = 9.41, p = 0.00, Cramér's V = 0.21 and avoiding both CL, χ²(1, N = 153) = 7.84, p = 0.00, Cramér's V = 0.23, and RS χ²(1, N = 153) = 14.38, p = 0.00, Cramér's V = 0.31 for vision correction. Also, suggestions from friends or family played a big role in choosing eyeglasses χ²(7, N = 153) = 6.71, p = 0.00, Cramér's V = 0.23, and in deciding to avoid CL, χ²(1, N = 153) = 4.95, p = 0.041, Cramér's V = 0.18, but it didn't impact the choice to avoid RS (p = 0.15). The recommendations from eye professionals significantly influenced the selection of eyeglasses χ²(1, N = 153) = 8.35, p = 0.00, Cramér's V = 0.32, but it did not show a significant influence for avoiding both CL (p = 0.36) and RS (p = 0.27).

**Table 2:** Participants satisfaction with glasses and reasons for preference and avoidance of alternative correction methods, (N = 153). Data are presented as frequency (N) and percentage (%). Significant at p < 0.05.

	Frequency (N)	Percentage (%)	χ²(df)	p-value
Reasons underlying the preference for eyeglasses				
Cosmetic preference	41	26.80%		
Cost considerations	72	47.10%	χ²(3) = 9.41	0.00*
Advice from friends or relatives	43	28.10%	χ²(7) = 6.71	0.00*
Recommendation by a professional	101	66.00%	χ²(1) = 8.35	0.00*
Work-related requirements	74	48.40%		
Advertising and promotional influence	9	5.90%		
Concerns about using contact lenses	74	48.40%		
Concerns about undergoing refractive surgery	58	37.90%		
Satisfaction with glasses use				
Strongly agree	40	26.10%		
Agree	70	45.80%		
Neutral	30	19.60%		
Disagree	9	5.90%		
Strongly disagree	4	2.60%		
Factors contributing to dissatisfaction with eyeglass use				
Cosmetic appearance	45	29.40%	χ²(2) = 36.37	0.00*
Sports limitations	46	30.10%		
Social perceptions	10	6.50%		
Discomfort	46	30.10%	χ²(2) = 15.61	0.00*
Frequent loss or breakage	44	28.80%		
Reasons for avoiding contact lenses				
Related side effects	107	69.90%		
Unfavorable professional recommendation	37	24.20%		
Unfavorable advice from friends or relatives	41	26.80%	χ²(1) = 4.95	0.04*
High cost	23	15.00%	χ²(1) = 7.84	0.00*
Difficulty with lens hygiene and care	84	54.90%		
Discomfort with touching the eye	78	51.00%		
Reasons for avoiding refractive surgery				
Concerns about undergoing refractive surgery	60	39.20%		
Insufficient information	61	39.90%		
Unfavorable professional recommendation	13	8.50%		
Unfavorable advice from friends or relatives	21	13.70%		
High cost	31	20.30%		
Negative publicity or reputation	28	18.30%	χ²(1) = 14.38	0.00*
Related side effects	70	45.80%		

The second group consisted of a total of 146 participants who used CL for vision correction, with 99.3% being females (N=145). The predominant age group was 20-25 years (95.9%, N = 140). Among them, 67.1% (N = 98) used soft contact lenses (SCL), and 32.9% (N = 48) used rigid gas permeable (RGP) lenses. Table 3 presents the reasons for this group's participants' preference for CL and their avoidance of the other options. Participants primarily preferred CL over the other corrective options for various reasons, such as cosmetic considerations (N = 120, 82.2%), inconvenience with glasses (n = 87, 59.6%), and work-related requirements (N = 70, 47.9%). In this group, 62.3% (N = 91) exhibited satisfaction with their CL, while only 9.6% (N = 14) reported dissatisfaction. Several factors contributed significantly to unhappy experiences with CL, including related side effects (N = 88, 60.3%, χ²(2, N = 146) = 20.93, p = 0.00, Cramér's V = 0.38), limitations of use during swimming (N = 82, 56.2%, χ²(2, N = 146) = 17.92, p = 0.00, Cramér's V = 0.35), difficulty with lens hygiene and care (N = 63, 43.2%, χ²(2, N = 146) = 15.93, p = 0.00, Cramér's V = 0.33), and high cost (N = 22, 15.1%, χ²(2, N = 146) = 13.51, p = 0.00, Cramér's V = 0.30). Participants were asked to provide reasons for avoiding eyeglasses: cosmetic appearance (N = 111, 76.0%), discomfort (N = 80, 54.8%), in addition to work-related requirements and frequent loss or breakage (N = 68, 46.6%). Additionally, participants avoided undergoing RS due to potential side effects (N = 81, 55.5%), a lack of sufficient information regarding the procedure (N = 70, 47.9%), and general concerns about undergoing the surgery (N = 56, 38.4%). In this group, cost considerations, advice from surroundings, and recommendations from eye care professionals did not influence the selection of CL and avoiding eyeglasses and RS for vision correction (p > 0.05).

**Table 3:** Participants satisfaction with contact lenses and reasons for preference and avoidance of alternative correction methods, (N = 146). Data are presented as frequency (N) and percentage (%). Significant at p < 0.05. CL: contact lenses.

	Frequency (N)	Percentage (%)	χ²(df)	p-value
Reasons underlying the preference for contact lenses				
Cosmetic preference	120	82.20%		
Cost considerations	57	39.00%		
Advice from friends or relatives	57	39.00%		
Recommendation by a professional	32	21.90%		
Work-related requirements	70	47.90%	χ²(48) = 91.44	0.64
Advertising and promotional influence	23	15.80%		
Discomfort with glasses	87	59.60%		
Concerns about undergoing refractive surgery	41	28.10%		
Sports suitability	37	25.30%		
Satisfaction with contact lenses use				
Strongly agree	39	26.70%		
Agree	52	35.60%		
Neutral	41	28.10%		
Disagree	9	6.20%		
Strongly disagree	5	3.40%		
Factors contributing to dissatisfaction with the use of CL				
Related side effects	88	60.30%	χ²(2) = 20.93	0.00*
High cost	22	15.10%	χ²(2) = 13.51	0.00*
Difficulty with lens hygiene and care	63	43.20%	χ²(2) = 15.93	0.00*
Limitations of use during swimming	82	56.20%	χ²(2) = 17.92	0.00*
Reasons for avoiding eyeglasses				
Cosmetic appearance	111	76.00%		
Unfavorable professional recommendation	15	10.30%		
Unfavorable advice from friends or relatives	6	4.10%		
Work-related requirements	68	46.60%		
Sports limitations	33	22.60%	χ²(35) = 61.84	0.95
Social perceptions	32	21.90%		
Discomfort	80	54.80%		
Frequent loss or breakage	68	46.60%		
Reasons for avoiding refractive surgery				
Concerns about undergoing refractive surgery	56	38.40%		
Insufficient information	70	47.90%		
Unfavorable professional recommendation	21	14.40%		
Unfavorable advice from friends or relatives	21	14.40%	χ²(47) = 59.14	0.54
High cost	32	21.90%		
Negative publicity or reputation	26	17.80%		
Related side effects	81	55.50%		

The third group consisted of a total of 152 participants who underwent RS to correct vision, with 32.9% (N = 50) being males (n = 50) and 67.1% (N = 102) being females. The main age group was 20-25 years (46.1%, N = 70), followed by the 25-30 years age group (32.9%, N = 50). Of those, 55.9% (n = 85) underwent LASIK, 42.8% (n = 65) underwent PRK, and 1.3% (n = 2) underwent Phemto-SMILE RS. Table 4 presents the reasons for this group's participants' preference for RS and their avoidance of alternative options. Participants primarily preferred RS over alternative corrective options for various reasons: discomfort with glasses and CL (n = 112, 73.7%), cosmetic preference (n = 102, 67.1%), and work-related requirements (n = 60, 39.5%). In this group, 88.8% (n = 135) exhibited satisfaction with their surgery results, while only 1.3% (N = 2) reported dissatisfaction. Several factors contributed significantly to the unhappy experience after the RS, including dryness (N = 24, 25.8%, χ²(3, N = 152) = 73.27, p = 0.00, Cramér's V = 0.69), visual side effects such as halos and glare (N = 17, 11.2%, χ²(3, N = 152) = 60.96, p = 0.00, Cramér's V = 0.63), and night vision problems (N = 4, 2.6%, χ²(3, N = 152) = 20.65, p = 0.00, Cramér's V = 0.37). Participants were asked to provide reasons for avoiding eyeglasses, including discomfort (N = 107, 70.4%) and cosmetic appearance (N = 103, 67.8%), in addition to work-related requirements (N = 63, 41.4%) and frequent loss or breakage (N = 60, 39.5%). Additionally, participants avoided CL due to their potential side effects (N = 102, 67.1%), challenges related to lens hygiene and care (N = 104, 68.4%), and discomfort associated with touching the eye (N = 90, 59.2%). In this group, the recommendation from eye care professionals significantly influenced the selection of RS χ²(1, N = 152) = 18.24, p = 0.00, Cramér's V = 0.26 as an option to correct vision and avoid both glasses, χ²(1, N = 152) = 22.08, p = 0.00, Cramér's V = 0.68, and CL χ²(1, N = 152) = 14.38, p = 0.00, Cramér's V = 0.38. While the advice from friends or relatives (p = 0.85) and cost considerations did not have a significant influence (p = 0.24).

**Table 4:** Participants satisfaction with refractive surgery and reasons for preference and avoidance of alternative correction methods, (N = 152). Data are presented as frequency (N) and percentage (%). Significant at p < 0.05. CL: contact lenses.

	Frequency (N)	Percentage (%)	χ²(df)	p-value
Reasons underlying the preference for refractive surgery				
Cosmetic preference	102	67.10%		
Cost considerations	47	30.90%		
Advice from friends or relatives	56	36.80%		
Recommendation by a professional	51	33.60%	χ²(1) = 18.24	0.00*
Work-related requirements	60	39.50%		
Advertising and promotional influence	25	16.40%		
Discomfort with glasses and CL	112	73.70%		
Sports suitability	48	31.60%		
Satisfaction with refractive surgery				
Strongly agree	106	69.70%		
Agree	29	19.10%		
Neutral	15	9.90%		
Disagree	2	1.30%		
Strongly disagree	0	0.00%		
Factors contributing to dissatisfaction with refractive surgery				
Visual side effects (halos, glare, etc.)	17	11.20%	χ²(3) = 60.96	0.00*
Night vision problems	4	2.60%	χ²(3) = 20.65	0.00*
Dryness	24	15.80%	χ²(3) = 73.27	0.00*
Reasons for avoiding eyeglasses				
Cosmetic appearance	103	67.80%		
Unfavorable professional recommendation	27	17.80%	χ²(1) = 22.08	0.00*
Unfavorable advice from friends or relatives	4	2.60%		
Work-related requirements	63	41.40%		
Sports limitations	46	30.30%		
Social perceptions	43	28.30%		
Discomfort	107	70.40%		
Frequent loss or breakage	60	39.50%		
Reasons for avoiding contact lenses				
Related side effects	102	67.10%		
Unfavorable professional recommendation	28	6.20%	χ²(1) = 14.38	0.00*
Unfavorable advice from friends or relatives	52	34.20%		
High cost	43	28.30%		
Difficulty with lens hygiene and care	104	68.40%		
Discomfort with touching the eye	90	59.20%		

A significant association between the method of refractive correction and the level of satisfaction was found, χ²(4, N = 451) = 28.52, p = 0.00. The highest satisfaction rate was found in the RS group (88.8%), followed by the glasses group at 71.9% and the CL group at 63.0%. Pairwise chi-square tests with a Bonferroni-adjusted alpha level (α = 0.017) showed no significant difference between the groups using glasses and CL, χ²(2, N = 299) = 2.75, p = 0.252. However, significant differences were noted between the RS group and the CL group, χ²(2, N = 298) = 28.64, p = 0.00, as well as between the RS group and the glasses group, χ²(2, N = 305) = 15.62, p = 0.00.

## Discussion

A significant rise in RE has been documented worldwide; efforts to address the RE should be prioritized since uncorrected RE accounts for approximately 50% of the moderate to severe visual impairment cases around the world, leading to a substantial decline in quality of life [1]. The current corrective methods include spectacles, CL, and RS [6], which individuals may select based on their preferences. This study demonstrates for the first time the factors influencing the patients' preferences among these different corrective methods.

The majority of participants are young, aged in their twenties, and exhibit mild to moderate myopia, which aligns with the global prevalence of RE [16]. The age, gender, education, and RE of the participants significantly influenced (p= 0.00) their choice of correction method, corroborating a prior study [17]. This indicates that various factors, including demographics and the severity of RE, play a role in this decision-making process.

In this study, participants reported multiple factors affecting their choice of corrective method, including peer influence, recommendations from eye care professionals, occupational needs, concerns about CL or RS, discomfort, cosmesis, and costs. In the first group, the choice of glasses was significantly affected (p = 0.00) by two primary factors: recommendations from professionals (66.0%) and cost (47.1%), alongside advice from friends or relatives (28.1%, p = 0.041). These findings highlight the state of spectacles as a practical, cost-effective, and clinically recommended option for vision correction. Although spectacles are commonly regarded as the most economical option [10], a prior study [18] indicated that the overall cost of spectacles may exceed that of CL or RS due to factors such as loss, breakage, and maintenance. Additionally, the cost of the spectacles is affected by the type of RE, with astigmatism being the most expensive, followed by hyperopia and myopia [18]. Notably, the majority of the current study sample had mild to moderate myopia, resulting in a decreased expense for the spectacle lenses.

CLs were not chosen by this group for several reasons, including concerns about potential side effects (69.9%), challenges with CL maintenance (54.9%), and discomfort associated with touching their eyes (51.0%). Similar reasons were reported previously [17,19-22]. These factors may lead CL users to discontinue their use and switch to spectacles as a more comfortable alternative option [19,22]. Moreover, these participants reported several factors for opting against RS, including concerns about possible side effects (45.8%), a lack of information about the procedure (39.2%), and general worries about the surgery (39.2%). These concerns are frequently documented in the literature [7,11,15,17,22,23]. Therefore, the preference for spectacles as a non-invasive option for correcting vision highlights a clear need for improved patient education by eye care practitioners regarding CL and RS options [13,24]. These efforts should focus on providing thorough information about the various available methods and emphasize that, with appropriate patient selection and compliance, the adverse effects can be minimized and effectively managed [13,24].

On the other hand, patients choosing CL as a corrective method indicated that cosmetic considerations (82.2%) and discomfort associated with glasses (59.6%) were the main motivators for their option. This aligns with an earlier study that identified inconvenience associated with glasses and enhanced cosmesis as the primary motivations for CL use across all age groups [7]. Considering that the majority of the study sample consists of females (76.7%), who typically exhibit greater concern for cosmetic appearance, particularly in the Middle Eastern region [14], this highlights that aesthetics play an important role in selecting the corrective method, especially among people concerned about appearance and those who find spectacles are impractical for their work environment, in addition to the further advantages CL offers, including a wider visual field and the capability to wear sunglasses. Additionally, 25.3% of this group reported that they opted for CL due to its suitability for sports, whereas 56.2% reported dissatisfaction with CL because of its limitations during swimming. Although CLs are favored over glasses in sports [8,9], this preference varies by sport type [8]. RS was avoided due to reasons similar to those reported by spectacle users, including potential side effects (55.5%), lack of adequate information about the procedure (47.9%), general concerns about the surgical process (38.4%), and cost considerations (17.8%). These concerns were reported previously in the literature [7,11,15,17,22,23], and indicate a necessity for extended doctor-patient counseling to thoroughly explain RS and enhance awareness about it.

The third group largely selected RS due to long-standing discomfort with glasses and CL (73.3%), cosmetic considerations (67.1%), and occupational practicality (39.5%). These findings support the results reported by a previous study [7] and suggest that aesthetic appearance has a great influence on decision-making, particularly for patients dissatisfied with the style of glasses. Additionally, 31.6% indicated a preference for RS due to its greater suitability for sports compared to spectacles and CL, confirming findings from prior research [7,8].

In this group, spectacles were avoided due to discomfort (70.4%) and unsatisfactory cosmetics (67.8%), as reported in previous research [7]. Additionally, 41.4% cited occupational requirements as a reason for their preference. CL were avoided for reasons comparable to those reported by the spectacle group: potential side effects (67.1%), maintenance challenges (68.4%), and discomfort with touching the eyes (59.2%). This indicates a progression in preference, with RS being recognized as the definitive solution for those seeking enhanced visual outcomes and physical comfort [14,15] who are dissatisfied with traditional options, despite RS's minimal side effects [11]. Although cost was not one of the primary factors for selecting RS and was reported as one reason for avoiding RS among the spectacles (20.3%) and CL (21.9%) groups, an earlier study indicated that RS is associated with lower annual expenses, as it involves a single payment and does not require lifelong expenses associated with loss, breakage of glasses, or maintenance of CL [18].

This study also showed that external influence from peers or eye care professionals has varying roles in the correction method. Advice from peers has a significant impact on the patient selection of glasses and avoidance of CL (26.8%, p = 0.041), but not on avoidance of RS selection (13.7%, p > 0.05). Conversely, the recommendations from eye care professionals showed a significant impact on the patient's selection of glasses (66.0%, p = 0.00), but they did not show a significant influence for avoiding both CL (p = 0.36) and RS (p = 0.27). Neither peers (39.0%) nor professionals (21.9%) significantly impacted the choice of CL or the decision to avoid other methods (p > 0.05). This finding is in contradiction with previous research [20,22], which indicated that recommendations from eye care practitioners influenced CL wearers, resulting in a cessation of CL use. The recommendations from eye care professionals showed a significant impact on the patient's selection of RS (33.6%, p = 0.00) where their advice significantly impacted (p = 0.00) the avoidance of glasses (17.8%) and CL (6.2%), while the advice from friends or relatives did not (p = 0.85). The results of the current study indicate that selection of the non-invasive option is affected by the patient's surroundings; CL is driven by a personal choice, while RS is largely influenced by professional recommendations.

A notable difference in satisfaction levels was observed among the three correction modalities, with the RS group exhibiting a significantly higher satisfaction rate (88.0%) when compared with the glasses group and the CL group (p = 0.00). The satisfaction rate of RS has been documented in prior research, ranging from 82% to 98% [24]. This figure suggests long-term satisfaction among participants who selected RS, likely attributable to the absence of daily visual aids, despite modest associated side effects that were also reported in the literature [11,15,24]. This suggests that appropriately selected patients could find the visual outcomes of RS highly successful and satisfying. The satisfaction level among eyeglasses users was quite high (71.9%), likely attributable to their practicality and cost-effectiveness [10]. However, dissatisfaction was significantly influenced (p = 0.00) by discomfort and sports limitations during wear (30.1%) in addition to cosmetic concerns (29.4%), as reported in previous research [7]. Suggesting that while spectacles provide a practical and cost-effective vision correction solution, physical discomfort and aesthetics remain as limitations affecting satisfaction. Although CLs offer superior aesthetic advantages, the CL group's satisfaction was the lowest among the three groups (62.3%), with dissatisfaction significantly influenced (p = 0.00) by related side effects (60.3%), limitation of use during swimming (56.2%), maintenance difficulties (43.2%), and cost (15.1%). These factors are consistent with prior research, which indicates that the main influences on satisfaction with the CL-wearing experience and dropout rates include CL-related ocular symptoms such as dryness and discomfort [19-22], maintenance difficulties [20,22], and cost [22,25]. Practitioners can mitigate CL cessation by addressing these factors early through careful monitoring, counseling, and effective patient education [22]. Overall, these findings indicate that satisfaction is influenced by a balance between the clinical outcomes and the limitations, emphasizing the importance of personalized decision-making.

While this study provided an insightful illustration of the factors influencing the patients' preference for vision correction methods, it has a few limitations. First, the majority of the samples are young and females, which restricts the generalization of the results of the findings. Additionally, satisfaction was self-reported by patients, making the data potentially susceptible to recall or cultural bias, where participants' memories or cultural influences may have affected their responses. The study did not also explore deeply into economic considerations nor include patients with complications from CL or RS, which may affect satisfaction and preferences. Moreover, due to the cross-sectional nature of this study, the findings are limited to identifying associations rather than establishing causal relationships between patient characteristics and satisfaction levels. Additionally, potential confounding variables, such as age, type of refractive error, and duration of use, were not statistically controlled, which may have influenced the observed associations. Future research may include a more diverse population and longitudinal follow-up to assess satisfaction over time. It could also investigate more in-depth the psychological, economic, and social factors that affect the patients' preferences, while incorporating the experiences of patients with adverse reactions.

## Conclusions

In conclusion, this study demonstrated that patients' selection of vision correction methods is influenced by various factors, including demographic variables, clinical information, peer and professional influence, practical needs, costs, and aesthetic considerations. While spectacles provide the most practical and economical alternative, CLs offer enhanced aesthetics, but a reduced satisfaction was reported due to accompanying side effects and maintenance difficulties. RS yielded the highest satisfaction levels, despite its associated side effects, among patients desiring freedom from daily visual correction and improved aesthetics. These findings reinforce the importance of tailored patient counseling and decision-making by taking into account not only clinical suitability but also individual behavioral features and lifestyle considerations.
